# Serum IgE Predicts Difference of Population and Allergens in Allergic Diseases: Data from Weifang City, China

**DOI:** 10.1155/2021/6627087

**Published:** 2021-06-25

**Authors:** Zhang Xu-De, Guo Bei-Bei, Wang Xi-Juan, Li Hai-Bo, Zhang Li-Li, Liu Feng-Xia

**Affiliations:** ^1^Department of Allergy, The First Affiliated Hospital of Weifang Medical University/Weifang People's Hospital, Weifang, China; ^2^Department of Central Laboratory, The First Affiliated Hospital of Weifang Medical University/Weifang People's Hospital, Weifang, China

## Abstract

**Background:**

Immunoglobulin E (IgE) is the most important promoter of allergic inflammation. However, there are few systematic studies on IgE in age range, genders, disease spectrum, and time regularity.

**Aim:**

To screen the common allergens, allergen spectrum, and IgE difference between type 2 inflammatory allergic diseases and other allergic diseases in Weifang, China.

**Methods:**

A retrospective study was performed by estimating patients' clinical data suffering from allergic diseases (urticaria, pollinosis, allergic rhinitis, atopic dermatitis, and bronchial asthma) between May 2019 and April 2020 using an allergen detection kit of Macro-Union Pharmaceutical.

**Results:**

732 of the 1367 patients showed different antigen positive, and the positive rate was 53.5%. The most common allergens were dust mites, mixed fungi, Artemisia pollen, cat/dog dander, and cockroaches. There were 27.0% (369/1367) of the patients with single positive allergen-specific IgE (sIgE), 26.5% (363/1367) with multiple-positive IgE. The total immunoglobulin E (tIgE) levels varied with gender, age, and type of disease. There was a difference in the distribution of allergens between children and adults. A positive correlation between the serum-specific IgE and the corresponding local inhaled allergen density was observed.

**Conclusions:**

In this study, we found that type 2 inflammatory allergic diseases have higher serum IgE and a higher probability of inhaled sIgE positive. According to age, gender, and condition, serological IgE detection of allergens provides new insight into the early diagnosis and prevention of allergic diseases.

## 1. Introduction

IgE-mediated allergic diseases are usually multisymptomatic, including allergic rhinitis (AR), allergic asthma (AS), urticaria, atopic dermatitis (AD), and eczema, which have become significant public health issues. The pathogenesis remains largely unknown. Allergic inflammation induced by certain inhaled substances or food antigens in the environment has been implicated in IgE-mediated allergic diseases. Based on the biological mechanisms that underline these diseases, AR, atopic dermatitis, and AS are widely regarded as classic type 2 inflammatory (Th2-dominated response) with the increase of circulating IgE level-eosinophilic inflammation in the human body [1–4]. Long sustained exposure to airborne allergens is known to result in persistent inflammation in AR and AS. Regarding urticaria, IgE-mediated mast cell activation, degranulation, and release of histamine and inflammatory mediators play critical roles in the pathogenesis of allergic diseases [5]. Many studies have suggested that IgE plays a crucial role in immune and inflammatory responses, which is a Th2 biomarker and participates in regulating Th2 inflammatory response.

The prevalence of allergic diseases has been raised due to increased environmental and industrial exposures in recent decades [6–8]. Environmental factors play an important role in the pathogenesis of AR and other respiratory and skin allergic diseases [9–11]. The prevalence of IgE-mediated allergic diseases increased progressively in the developed countries, which currently account for 10% of children subject to food allergy [12], and 40% of the population with allergic rhinitis [13, 14]. There are over 330 million asthma patients worldwide [15], which accounts for 20% of children and 2-18% of adults among the AD population [16]. However, up to now, the high prevalence of allergic diseases in the population has not been effectively curbed, and people are still plagued by diseases, which indicates that the human understanding of the diagnosis and treatment of such diseases is still insufficient [17–19]. The increased global prevalence of allergic diseases is mainly attributed to environmental factors, suggesting that controlling environmental exposures may protect against allergic diseases in predisposed individuals. Therefore, it is of great significance to identify allergens for the prevention and treatment of allergic diseases. With industrialization development in China, allergic diseases have become a public health concern with increasing incidence. The prevalence of allergic disorders is closely related to various environmental allergens implicated in AR and asthma, including dust mite, mold, pollen, and animal fur [20, 21]. Although the prevalence and possible causes of AR/AS have been well documented in many developed countries, little information is available in China [22]. Because of the vast territory, different topographic, climatic, and economic conditions, and plant species, allergen spectrum is different from region to region in China. In view of this, we collect data of 1367 patients with allergic diseases, including AS, AR, AD/eczema, and urticaria. In this study, we aim to explore the allergen spectrum of Weifang city in China and investigate the association between allergic reaction and specific allergens, which will thus provide a rationale for the selecting allergens to be tested based on the clinical presentations.

## 2. Methods

### 2.1. Study Subjects

This retrospective study was approved by the Ethics Review Board of Weifang People's Hospital, Weifang, China. A total of 79 nonatopic subjects and 1367 patients with AR, AS, AD, and urticaria, who received treatment at the Department of Allergy, Respiration and Pediatrics in Weifang People's Hospital between May 2019 and April 2020, were enrolled in the study. Only the first test report was included if the same person met the diagnostic allergen detection multiple times within the same time range. Patients with ambiguous and suspicious diagnoses were excluded. There were 638 males and 729 females aged from 2 months to 87 years old. There were 266 infants aged 0-4 years, 341 school-age children (5-11 y) and adolescents (12-17 y), and 760 adults aged over 18 years. Four hundred four cases of urticaria, 345 cases of AD and eczema, 324 cases of AS and cough variant asthma (CVA), and 233 patients of AR (including pollinosis) were all included (Table 1).

### 2.2. Allergen-Specific IgE Antibody Detection

2-4 ml fresh peripheral venous blood was collected and clotted at room temperature for 20 minutes, centrifuged at 3600 rpm for 4 minutes. The serum was separated and stored at 4°C for examination. The inhaled allergens and ingested allergens were tested using an allergen sIgE antibody detection kit (Beijing Macro-Union Pharmaceutical Co. Ltd., China). The allergen-specific IgE antibody detection kit was used to detect serum sIgE by chemiluminescent immunoassay, whose clinical usefulness was documented compared to other assays for specific IgE measurement [23]. A standard curve was established for quality control. Briefly, 96-well plates coated with allergen were put into the CLIA200 automatic operation platform. 50 *μ*l of the sample diluent was added into each well. 50 *μ*l of the sample was added to the mix. The coated plate was sealed with the sealing film and incubated in a 37°C constant temperature incubator for 45 minutes. After incubation, the vessel was diluted and released. The working solution shall not be less than 350 *μ*l for washing, which shall be left for 30 s and pat dry for five times in total. After washing the plate, we added 100 *μ*l enzyme conjugates to each well and passed the plate after incubation. After that, 50 *μ*l luminescent substrate A and substrate B were added into each well, respectively. The vessel was shaken at room temperature for 5 minutes, avoiding light. The luminescent intensity (RLU) of each pore was measured by chemiluminescent immunoassay (Yantai Addcare Bio-Tech Co. Ltd., China). The double logarithm fitting software was used for analysis.

### 2.3. Diagnosis of Allergic Diseases

In this study, we have defined different allergic diseases simply and effectively according to the international consensus of diagnostics so that researchers can screen them from thousands of medical records: AR: rhinorrhea, nasal obstruction, nasal itching, and sneezing with or without ocular symptoms [24]; AS: recurrent episodes of wheeze, cough, breathlessness, and chest tightness, which are usually associated with variable airflow obstruction and bronchial (airway) hyperresponsiveness [25]; AD: xerosis, pruritus, and erythematous lesions with increased transepidermal water loss [26]; urticaria: the sudden appearance of wheals and/or angioedema, those with a course of more than six weeks are defined as chronic urticaria (CU), or acute urticaria [27, 28]; eczema: clusters of punctate erythema and needle to miliary-sized papules and papules and herpes, dense patches, basal flush, mild swelling, diffuse boundary, dry skin, and severe itching [29]; papular urticaria: typical local or systemic red papules with constant itching; anaphylactic reaction: the severe allergic reaction that is rapid in onset and may cause death including difficulty in breathing and chest tightness, laryngeal edema, and anaphylactic shock [30].

### 2.4. Statistical Analysis

SPSS version 19.0 software (SPSS Inc., Chicago, IL, USA) was used for data analysis. Positive rate (%) is the percentage of the positive serum test to various allergens. Different diseases are considered as categorical variables. The chi-square test evaluates the difference among variables. A two-tailed *p* < 0.05 is considered to be statistically significant.

## 3. Results

### 3.1. Age, Gender, and Serum tIgE in All Participants

1367 patients and 79 nonatopic subjects were included in the study. The patients' mean age was 25.1 years (95% CI, 24.0 to 26.1 years). There were 267, 338, and 762 patients in the 0-4 (infancy), 5-17 y, and adult groups. The tIgE of 934 patients (68.3%) exceeded 100 IU/ml. The mean value of tIgE was 225.4 IU/ml in males and 192.3 IU/ml in females. There was a significant difference in tIgE between groups regarding gender (*p* < 0.001) (Table 2).

**Figure 1:**
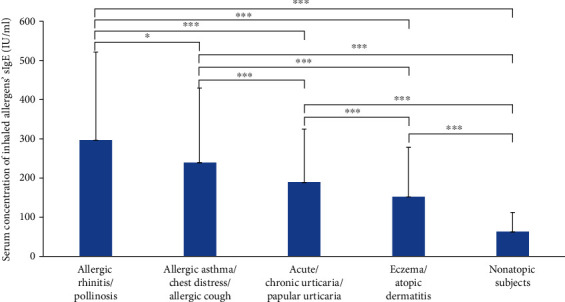
Mean value of total IgE in different diseases (^∗∗^*p* = 0.024, ^∗∗∗^*p* < 0.001).

The mean of tIgE in the 0-4 age group was 190.6 IU/ml (95% CI, 173.9 to 207.4 IU/m). The mean value of tIgE in the 5-17 groups was 252.0 IU/ml (95% CI, 229.7 to 274.4 IU/ml). The mean value of tIgE in the adult groups was 194.1 IU/ml (95% CI, 182.4 to 205.8 IU/m). The difference of tIgE was significant between the adolescent and adults (*p* < 0.001). Similar results were found between the infant group and the adolescent group (*p* < 0.001). There were 544 cases of hypersensitivity of the respiratory system. The average total IgE was 263.3 IU/ml. The tIgE of 828 patients only with cutaneous symptoms was 171.7 IU/ml. When we further refined the disease into AR, AS/cough/chest distress, acute/chronic urticaria, and eczema/AD (complications excluded), the average tIgE was decreased in turn, and the differences between groups were statistically significant (Figure 1).

### 3.2. Average Age Associated with Single Positive Allergen in Allergic Disease Patients

As shown in Table 3, the number of allergens of allergic disease patients was as follows: dust mite (394), mixed fungi (266), Artemisia pollen (173), Humulus scandens pollen (123), cockroach (109), dog dander (104), cat dander (89), ragweed pollen (77), milk (49), egg white (42), soybeans (40), tree pollen (poplar/willow/elm) (36), sea fish/crab (30), peanuts (23), and sea shrimp (19). As a result, it could be quickly concluded that dust mites, mixed fungi, Artemisia pollen, and Humulus pollen were the main allergens causing allergic diseases in Weifang, China.

**Figure 2:**
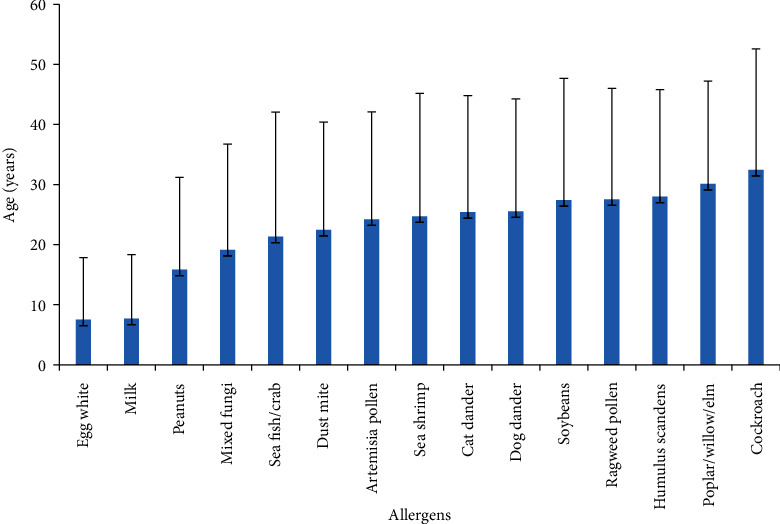
Average age of single allergen sensitization.

The age of the allergic population varied with different allergens: egg: 7.5 years (95% CI, 4.3 to 10.7 years), milk: 7.7 years (95% CI, 4.7 to 10.7 years), peanut: 15.8 years (95% CI, 9.2 to 22.5 years), mixed fungi: 19.1 years (95% CI, 16.9 to 21.3 years), dust mite: 22.4 years (95% CI, 20.6 to 24.3 years), Artemisia pollen: 24.2 years (95% CI, 21.4 to 27.0 years), cat: 25.4 years (95% CI, 21.3 to 29.5 years), dog: 25.5 years (95% CI, 21.8 to 29.2 years), Humulus scandens: 27.7 years (95% CI, 24.4 to 30.9 years), tree pollen: 30.1 years (95% CI, 24.3 to 35.9 years), soybean: 27.4 years (95% CI, 20.9 to 33.9 years), ragweed pollen: 27.5 years (95% CI, 23.1 to 31.9 years), and cockroach: 32.4 years (95% CI, 28.6 to 36.2 years) (Figure 2).

### 3.3. sIgE Level of Inhaled Allergens and Ingestible Allergens in Different Allergic Disease Patients

**Figure 3:**
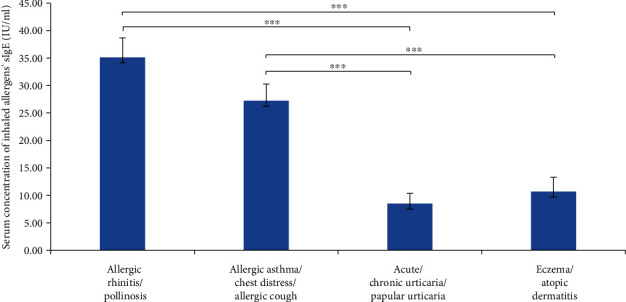
Mean value of inhaled allergen's sIgE in different diseases (^∗∗∗^*p* < 0.001).

**Figure 4:**
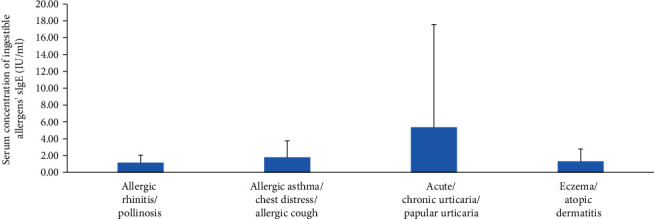
Mean value of ingestible allergen's sIgE in different diseases.

The level of inhaled sIgE was significantly different between the respiratory allergic disease and the allergic skin disease (*p* < 0.001). In contrast, no statistical differences of sIgE were found between groups of AR and AS, urticaria, and eczema. There was no significant difference of sIgE between the two groups of inhaled allergens and ingestible allergens. Moreover, patients with respiratory allergies had higher levels of sIgE than those with allergic dermatosis, and most patients with respiratory allergies were aeroallergens positive (Figures 3 and 4). There was no significant correlation between tIgE and sIgE in all allergic groups.

### 3.4. Time Regularity of Allergic Disease Positive with Specific Allergens

**Figure 5:**
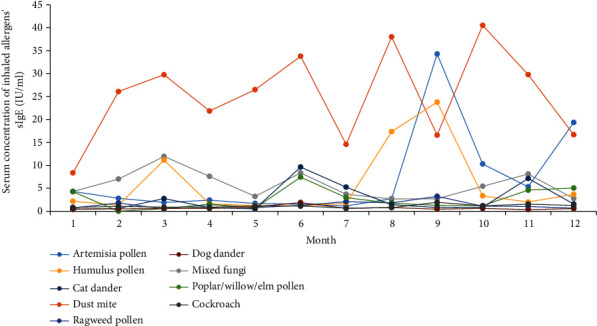
sIgE levels of 9 inhaled allergens in different months.

**Figure 6:**
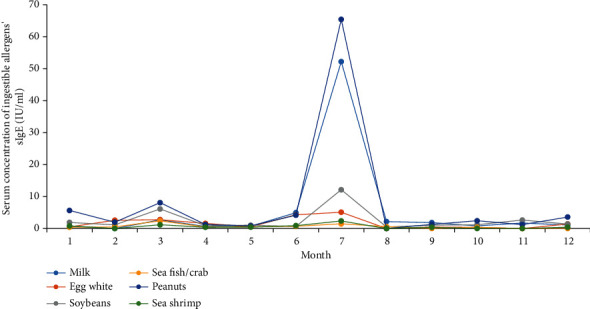
sIgE levels of 6 ingestible allergens in different months.

The median concentration of sIgE in patients positive with 15 specific allergens in different months of the year was analyzed. There was a peak in March, June, and November for dust mite and mixed fungi, respectively. Also, patients' sIgE level positive with dust mite peaked in August, while Artemisia and Humulus pollen had only one peak in September. The peak of poplar/willow/elm's sIgE appeared in June, while the annual dispersal of tree pollen occurred in May. Accordingly, as reported in the previous study, there was one month time postponement between the air concentration of tree pollen and the corresponding serum sIgE concentration of patients [31]. A positive correlation was found between annual total pollen quantity and median sIgE values (Figure 5). In contrast, the incidence of ingestible allergens, especially regarding peanuts, soybeans, and milk, was most common in March and July (Figure 6).

## 4. Discussion

More and more epidemiological studies have confirmed the objective existence of “atopic march”; consequently, the early prevention and prediction of allergic diseases have become a hot issue [32–35]. Numerous studies have confirmed that antigens can sensitize the body through the damaged epidermal layer, thereby initiating local and systemic immune responses, increasing the incidence of eczema, AR, and AS [36]. It has been proposed that the observed temporal relationship between atopic diseases may help earlier diagnosis and may facilitate novel approaches to prevent the disease [34, 37]. This study found that the primary manifestation of allergic infants and young children is AD caused by ingestible allergens. Allergic adolescents mainly have respiratory disorders. The sIgE of dust mites and various airborne pollen is gradually detected, which can change with age. Recently, Belgrave et al. concluded that the developmental profiles of eczema, wheeze, and rhinitis were heterogeneous, and only a tiny proportion of children (7% of those with symptoms) followed trajectory profiles resembling the atopic march [38]. Nevertheless, each region has its unique allergen spectrum due to differences in heredity, diet, and lifestyle. Zhang and Zhang [39] reviewed the sensitization pattern to inhalant allergens among AR patients in the mainland of China. They found that the prevalence and type of aeroallergens were different in diverse cities and regions. This study investigates the disease spectrum of allergic diseases according to the other areas and allergens by pooling data from 1367 patients with allergic diseases.

Although previous studies have shown the association of serum tIgE and sIgE levels with allergic diseases, few studies address this association in general. We have found that the mean tIgE in males was higher than that in females (*p* < 0.05). We hypothesize that sex hormone may affect the level of serum IgE. In terms of age, the concentration of tIgE is observed to increase from birth to adolescence and then decrease during adulthood, which has been validated in a previous study [40, 41]. It has been suggested that the level of tIgE in Th2-driven respiratory inflammatory diseases is significantly higher than that in inflammatory skin diseases. Therefore, tIgE has developed as a Th2 biomarker, which is involved in regulating Th2 inflammatory response. The determination of serum tIgE in asthma patients can help determine the disease's severity, thus guiding the acute attack period's treatment and preventing remission [42].

The main allergens of respiratory allergy are dust mites, mixed fungi, Artemisia pollen, and Humulus scandens pollen. Dust mites are the main allergens in north China, consistent with other reports [39, 43]. Limited by the types of allergens detected in the study, we do not have the data of the most common allergens in spring, such as Platanus orientalis and ash tree pollen. Therefore, a large part of AR and pollinosis patients with negative sIgE and high tIgE in spring are diagnosed by the skin prick test (SPT). At the same time, poplar/willows, which are native trees of north China, have visible white floccules but are rarely sensitized. For inflammatory skin diseases, urticaria accounts for 29.6% of the allergic population in this study, and 37% of them are detected as allergen-positive. Although the etiology of urticaria is complex, many relevant causes and/or triggers have been discovered, such as food in infants and children [44]. We have found that ingestion of many foods, including eggs, milk, and peanuts in sensitized infants/children, may cause acute urticaria more frequently than in adults. In adult urticaria, the IgE antibody in blood is more likely to be specific for inhalant allergens, such as dust mites and airborne pollen.

In this study, 87% of allergen-related eczema appeared after childhood. Food sensitization was only present in 21% of patients. 58% of the eczema population had more than one allergen. We also observed some IgE sensitization patterns, mainly including dust mites and molds, associated with persistent eczema/allergic dermatitis, especially in adults. The main food allergens for infantile eczema were still eggs and milk. Sensitization to sea fish/crab, sea shrimp, and peanuts among atopic children in Weifang was rarely observed. Although allergic patients will be refrained from eating spicy foods and seafood, we hypothesize that a non-IgE-mediated pathway may cause aggravation of dermatitis symptoms after seafood intake. Attributed to the gene differences with western populations, peanut and soybean nuts are not the leading causes of allergy in the Chinese population.

Among all immunoglobulin subclasses, IgE stands out concerning its low serum concentration and short half-life of 2 days. Seasonal allergen exposure can induce a rapid increase in allergen-specific IgE levels [45, 46]. This study analyzed the time regularity of serum sIgE concentration of four main allergens, including dust mite, mixed fungi, Artemisia pollen, and Humulus pollen. There is a 1 month postponement between the serum sIgE concentration peak and the corresponding plant pollens' seasonal dissemination peak [47, 48]. Although the environmental density data of dust mites and fungi are insufficient in Weifang, the role of meteorological factors in the survival and reproduction of fungi and dust mites is critical [49, 50]. We speculate that the serum concentration of dust mites and fungi is closely related to seasonal reproduction. Therefore, it is necessary to detect airborne pollen and fungi as one of the routine meteorological forecast items and guide the allergic population to prevent and/or relocate to an allergen-free area for seasonal allergens.

Besides, sIgE levels of dust mites in inflammatory skin diseases (urticaria, eczema), classical type 2 inflammatory allergic diseases (AR and AS) are all statistically significant. The results have shown the allergic levels of dust mites in allergic skin patients were lower than those in respiratory allergic patients (*p* < 0.05). Similar findings have been found about fungi. There is a saying in China, namely, “one respiratory tract, one disease.” We have found that AR patients tend to have higher sIgE antibodies than those with AS, especially in the two significant allergens of dust mites and Artemisia pollen. Recent cross-sectional studies have shown that the same individual can display different inflammation profiles in various respiratory tract sites [51]. There is an issue of whether each allergen family should be given a different cut-off. In addition to the most critical four allergens causing allergies, airborne pollen concentrations and food allergen antibodies are lower. However, their role in the allergy process is undeniable. This confusion was particularly evident after clinical verification of SPT.

Our study has some limitations. First, the patient's information is not further refined, including asthma, rhinitis, dermatitis symptom scores, and other indicators to assess the disease's severity. Change in IgE concentration with seasons has not been accurately counted. Second, the number of patients in each group is different. Almost half of the patients are diagnosed with urticaria and patients with AD account for only 3.2% of the sample. Third, the reagent kit does not contain the most common inhaled allergens of the Platanus orientalis in the north of China in spring and the ingestive allergens of wheat. Finally, because most of the patients are referred from Weifang, IgE's disease entity and measurements may not represent atopic patients' general population in Shandong province. More studies are warranted to elucidate the role of serological IgE detection of allergens according to different age, gender, and other factors in allergic diseases.

## Figures and Tables

**Table 1:** Number and percentage of allergic diseases among 1367 patients.

Diseases	Number (proportions)
Urticaria (acute/chronic)	404 (29.6%)
Allergic dermatitis/eczema	345 (25.2%)
Allergic asthma/CVA/chest tightness	324 (23.7%)
Allergic rhinitis/pollinosis	233 (17.0%)
Anaphylactic reaction without clear predisposing factors	16 (1.2%)
Papular urticaria	30 (2.2%)
Allergic conjunctivitis	7 (0.5%)
Others	22 (1.6%)

**Table 2:** Characteristics of all participants regarding age and tIgE.

	Nonatopic subjects			Patients		
	Male	Female	Total	Male	Female	Total
Patients, no. (%)	37 (46.8)	42 (53.2)	79 (100)	638 (46.7)	729 (53.3)	1367 (100)
Age range (year (minimum-maximum))	7-75	4-76	4-76	0.3-86	0.4-87	0.3-87
Mean age (y (95% CI))	41.7 (36.0-47.5)	36.6 (30.6-42.5)	38.2 (34.0-42.5)	20.0 (18.5-21.6)	29.2 (27.8-30.6)	25.1 (24.0-26.1)
Mean total IgE (IU/ml (95% CI))	64.6 (45.7-83.5)	63.4 (51.5-75.3)	64.0 (53.3-74.6)	225.4 (211.4-239.5)	192.3 (182.2-204.4)	207.8 (198.5-217.0)
Sensitization to respiratory allergens, no. (%)	0 (0)	2 (4.8)	2 (2.5)	341 (53.4)	323 (44.3)^a^	664 (48.6)
Sensitization to ingestible allergens	0 (0)	0 (0)	0 (0)	89 (13.9)	63 (8.6)^b^	152 (11.1)

Chi-square test: ^a^*p* = 0.001; ^b^*p* = 0.002.

**Table 3:** Number of allergen-positive patients with the relevant allergic diseases.

	Ar	Mite	Dog	Cat	Cr	Ra	Hu	Fungi	Tree	Egg	Milk	Peanut	Soy	Shrimp	Fish
^∗^Allergic rhinitis/pollinosis	48	117	22	19	13	22	33	58	6	0	5	1	7	1	1
^∗^Allergic asthma/allergic cough/chest tightness	61	130	30	23	28	22	33	95	10	8	7	6	5	1	3
^∗^Acute/chronic urticaria, papular urticaria	26	83	25	23	33	20	26	54	14	15	19	11	14	6	9
^∗^Eczema/allergic dermatitis	30	60	26	24	33	10	25	59	5	19	18	4	12	11	16
Anaphylactic	8	4	1	0	2	3	6	0	1	0	0	1	2	0	1

Cr: cockroach; Hu: Humulus scandens; Ra: ragweed pollen. ^∗^Jonckheere-Terpstra test: *p* = 0.229.

## Data Availability

Data in this study is available from the corresponding author upon request.
